# Supplementary material for: Diagnostic Accuracy and Interrater Agreement of FDG-PET/CT Lymph Node Staging in High-Risk Endometrial Cancer: The SENTIREC-Endo Study
Source: Cancers (Basel). 2025 Jul 19;17(14):2396. doi: 10.3390/cancers17142396 (PMC12293347; doi:10.3390/cancers17142396)
Supplement: Supplementary file 1 [file cancers-17-02396-s001.zip › cancers-3722605-supplementary.pdf]

Table S1: Inclusion and exclusion criteria in the SENTIREC-endo high-risk endometrial cancer study.

Inclusion criteria	Exclusion criteria
• Women 18 years or older<br>• Diagnosed with expected endometrial adenocarcinoma (EAC) FIGO I high risk histology (grade 3 endometriod adenocarcinoma, serous-, clear cell, or de-differentiated adenocarcinoma, or carcinosarcoma)<br>• Triaged for robot assisted surgery<br>• Can understand and fill out questionnaires in Danish<br>• Have had an up-front FDG-PET/CT performed as the diagnostic workup	• Women who formerly had radical pelvic lymph node dissection performed<br>• Known allergy to the sentinel node procedure solution for injection; "Indocyanine Green" or "Verdye"), or known allergy to iodide (5 % iodide can be contained in mentioned solutions)<br>• Women where treatment with radical pelvic lymph dissection have been deselected for other reasons (e.g. comorbidity or suspected other risk factors to the procedure)<br>• Women in active treatment for other cancers in the last 5 years (not including basal cell carcinoma)<br>• Patients with known dementia<br>• Women included in other studies that can influence the outcome of the SENTIREC-endo study.
